# TCS2 Increases Olaquindox-Induced Apoptosis by Upregulation of ROS Production and Downregulation of Autophagy in HEK293 Cells

**DOI:** 10.3390/molecules22040595

**Published:** 2017-04-07

**Authors:** Daowen Li, Kena Zhao, Xiayun Yang, Xilong Xiao, Shusheng Tang

**Affiliations:** College of Veterinary Medicine, China Agricultural University, Yuanmingyuan West Road No. 2, Haidian District, Beijing 100193, China; lidaowen123.fff@163.com (D.L.); zkncau@163.com (K.Z.); fujianlw@163.com (X.Y.); xiaoxilong1958@163.com (X.X.)

**Keywords:** olaquindox, TSC2, autophagy, cytotoxicity, oxidative stress

## Abstract

Olaquindox, a feed additive, has drawn public attention due to its potential mutagenicity, genotoxicity, hepatoxicity and nephrotoxicity. The purpose of this study was to investigate the role of tuberous sclerosis complex (TSC2) pathways in olaquindox-induced autophagy in human embryonic kidney 293 (HEK293) cells. The results revealed that olaquindox treatment reduced the cell viability of HEK293 cells and downregulated the expression of TSC2 in a dose- and time-dependent manner. Meanwhile, olaquindox treatment markedly induced the production of reactive oxygen species (ROS), cascaded to autophagy, oxidative stress, and apoptotic cell death, which was effectively eliminated by the antioxidant *N*-acetylcysteine (NAC). Furthermore, overexpression of TSC2 attenuated olaquindox-induced autophagy in contrast to inducing the production of ROS, oxidative stress and apoptosis. Consistently, knockdown of TSC2 upregulated autophagy, and decreased olaquindox-induced cell apoptosis. In conclusion, our findings indicate that TSC2 partly participates in olaquindox-induced autophagy, oxidative stress and apoptosis, and demonstrate that TSC2 has a negative regulation role in olaquindox-induced autophagy in HEK293 cells.

## 1. Introduction

Olaquindox, a quinoxaline-*N*,*N*-dioxide (QdNO), has been used as a therapeutic feed additive for improving the feed efficiency and controlling dysentery in food-producing animals [1]. Despite the fact that olaquindox is a good antibacterial agent and growth promoter, its use has been forbidden or restricted due to its genotoxicity [2], hepatoxicity [3] and mutagenicity [4]. Olaquindox could apparently affect human health due to potentially toxic residues in edible animal-origin products. In long-term toxicity studies of olaquindox in rats it has been demonstrated that toxic effects were detected in the kidney, liver and endocrine glands [5]. According to a new study, even at a relatively low concentration of olaquindox (6.6 μg/mL), significant mutagenic effects may result and the mutation frequency was increased by up to 12-fold [6].

Oxidative stress damage, caused by excessive reactive oxygen species (ROS), has been suggested as a plausible mechanism for QdNO-induced toxicity and metabolism studies suggest that oxidative stress plays a critical role in QdNOs-induced cytotoxicity [7]. It has been inferred that the genotoxic effects caused by olaquindox were perhaps due to ROS-induced oxidative DNA damage in human hepatoma G2 (HepG2) cells [3]. Zhao et al. have proposed that olaquindox treatment could trigger intracellular ROS production and lead to the activation of MAPK pathways involved in the regulation of apoptosis in HepG2 cells [8]. In our previous studies, olaquindox directly induced ROS generation and knockdown of GADD45a further aggravated ROS-mediated apoptosis [9].

Recent observations have indicated that olaquindox could induce autophagy in HepG2 cells [10]. Autophagy, a primary metabolic process by which eukaryotic cells degrade, is essential for cellular integrity and intracellular homeostasis, cell survival and growth [11]. During this process, substances in the cytoplasm are phagocytosed by autophagosomes, which are spherical structures with double layer membranes and are transported to the lysosomes for degradation [12]. Actually, convincing evidence has suggested a close interaction between apoptosis and autophagy [13]. Recent research showed that autophagy and apoptosis share similar effectors and regulators, which are induced by the same stimuli [14]. Data demonstrate that inhibition of autophagy promoted tunicamycin-induced apoptosis in HepG2 cells [15]. Furthermore, it has been reported that colistin induced caspase-dependent apoptosis and autophagy in neuronal cells, involving ROS-mediated oxidative stress [16]. In addition, a previous study has pointed out that quinocetone-induced autophagy was mediated by AKT/TSC2/p70S6K signaling pathway, and inhibition of autophagy promoted quinocetone-treated cell survival by attenuating apoptosis [17]. This indicated that TSC2 may play a critical role in autophagy signaling pathway. Tuberous sclerosis complex (TSC), a negative regulator of mTOR pathway, is a genetic disease characterized by benign tumors in various organs [18]. It is suggested that autophagy through the TSC2-mTOR pathway plays a critical role in maintaining the cardiac function and quantity of mitochondria [19]. A study has shown that the complex activity of neuronal TSC1/2 was essential for the coordinated regulation of autophagy through AMPK pathway [20]. In our previous study, we have demonstrated that quinocetone-induced autophagy was mediated by the TSC2 signaling pathway, and inhibition of autophagy promoted quinocetone-treated cell survival [17]. In this study, we aimed to provide a model of the in vitro toxicology of olaquindox in HEK 293 cells. What’s more, we explored the effect of TSC2 in olaquindox-induced autophagy. Our findings should contribute to the understanding of the molecular mechanisms of olaquindox toxicity.

## 2. Results

### 2.1. Effect of Olaquindox on HEK293 Cell Viability and Apoptosis

**Figure 1:**
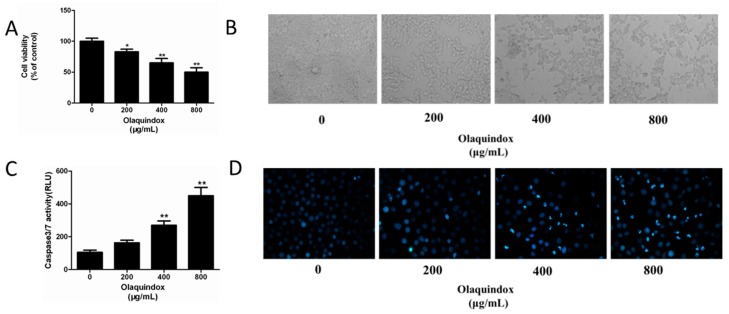
Effect of olaquindox on HEK293 cell viability and apoptosis. (**A**) Cell viability of 293 cells was estimated by MTT assays. Viability of control cells was set as 100%; (**B**) 293 cells were observed under a Leica inverted light microscope (400×); (**C**) Cells were exposed to specified concentrations of olaquindox for 24 h. Caspase activities were separately determined by the Caspase-Glo^®^ 3/7 assay kit; (**D**) Cells stained with Hoechst 33342 were observed under an inverted fluorescence microscopy (400×). All data represents means ± SD from three independent experiments. * *p* < 0.05, ** *p* < 0.01, compared to the control group.

The cytotoxicity to HEK 293 cells exposed to olaquindox for 24 h was examined. As shown in Figure 1A, the cell viability was significantly reduced in a dose-dependent manner after olaquindox treatment, with an IC_50_ (inhibitory concentration 50%) of 800 ± 24.5 μg/mL.

Morphological observations suggested that olaquindox treatment induced cell shrinkage, detachment from neighboring cells and cytoplasmic extensions, which reduced cell viability (Figure 1B). As shown in Figure 1D, compared with the control, treatment of HEK293 cells with 800 μg/mL olaquindox for 24 h led to the shrinkage of nuclei and condensation of chromatin, followed by the appearance of apoptotic bodies. Meanwhile, the activity of caspase-3/7 in HEK293 cells was significantly increased after exposure to olaquindox for 24 h (Figure 1C).

### 2.2. Effects of Olaquindox on ROS Generation and Oxidative Stress in HEK293 Cells

**Figure 2:**
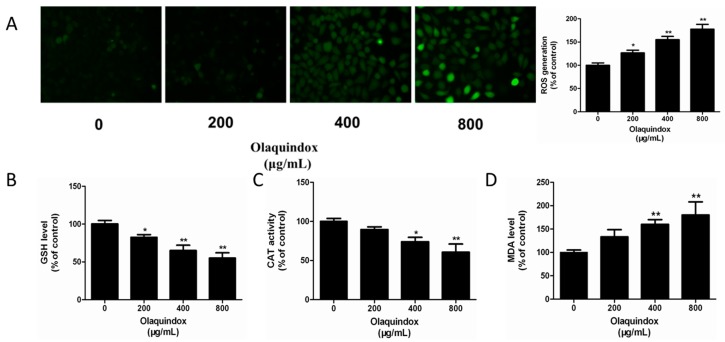
Effects of olaquindox on ROS generation and oxidative stress in HEK293 cells. Cells were treated with specified concentrations of olaquindox for 24 h. (**A**) Intracellular ROS production was measured by 2,7-dichlorofluorescein diacetate (DCFH-DA) and observed under a Leica inverted fluorescence microscope (400×). ROS produced relative to control were quantified; (**B**–**D**) Effect of olaquindox treated on GSH levels, CAT activities and MDA levels, respectively. All data represents means ± SD from three independent experiments. * *p* < 0.05, ** *p* < 0.01, compared to the control group.

Biomarkers of oxidative stress including cellular glutathione (GSH), catalase (CAT) and malondialdehyde (MDA) and ROS production were detected. In the groups treated with 400 and 800 μg/mL olaquindox, the results showed that intracellular ROS generation was significantly enhanced (*p* < 0.01) (Figure 2A). Meanwhile, GSH levels were significantly reduced to 68.4% and 56.2% (*p* < 0.01) (Figure 2B) and CAT activity was significantly decreased to 75.6% and 63.8% (*p* < 0.01) (Figure 2C). Compared to the control, exposure to 400 and 800 μg/mL olaquindox dramatically increased the MDA levels to 161.2% (*p* < 0.01) and 189.48% (*p* < 0.01) (Figure 2D).

### 2.3. Effect of Olaqindox on Autophagy in HEK293 Cells

**Figure 3:**
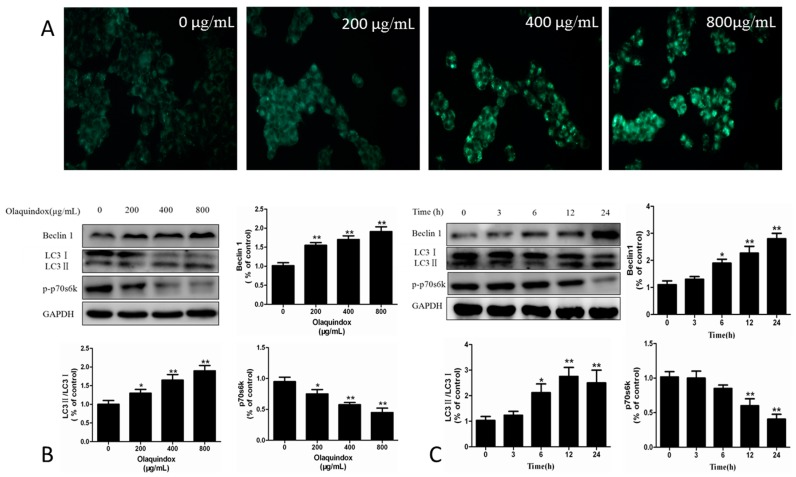
Effect of olaquindox on autophagy in HEK293 cells. (**A**) Autophagic vacuoles induced by olaquindox were stained with monodansylcadaverine (MDC). Cells were treated with olaquindox for 24 h, and then incubated with medium containing 50 μM MDC for 30 min in the dark at 37 °C. Cells were observed under a Leica inverted fluorescence microscope (400×); (**B**) Expression of Beclin 1, LC3II/LC3I and p70s6k treated with olaquindox (0, 200, 400, 800 μg/mL) for 24 h were detected by western blotting analysis, GAPDH was used for loading control; (**C**) Cells treated with 800 μg/mL olaquindox for different time points (0–24 h) and expression of Beclin 1, LC3II/LC3I and p70s6k were detected by western blotting analysis. All data represents means ± SD from three independent experiments. * *p* < 0.05, ** *p* < 0.01, compared to the control group.

Monodansylcadaverine (MDC) staining is used to detect the formation of acidic vesicular organelles. As shown in Figure 3A, after olaquindox treatment for 24 h, the percentage of autophagic HEK293 cells increased in a dose-dependent manner. To further confirm olaquindox-induced autophagy, we detected the expression of autophagy marker proteins like LC3, Beclin 1 and phosphorylation-p70s6k. After olaquindox treatment for 24 h, compared with the control group, the expression of Beclin 1 (~1.8-fold), LC3II/LC3I (~1.9-fold), all significantly increased in the 800 μg/mL olaquindox group (both *p* < 0.01) (Figure 3B). However, the expression of phosphorylation-p70s6k decreased to 0.62-fold and 0.45-fold (both *p* < 0.01) in the olaquindox 400 and 800 μg/mL groups. This indicated that olaquindox could induce autophagy in HEK293 cells (Figure 3C).

### 2.4. Effect of Reduced ROS Level on Olaquindox Induced Autophagy

**Figure 4:**
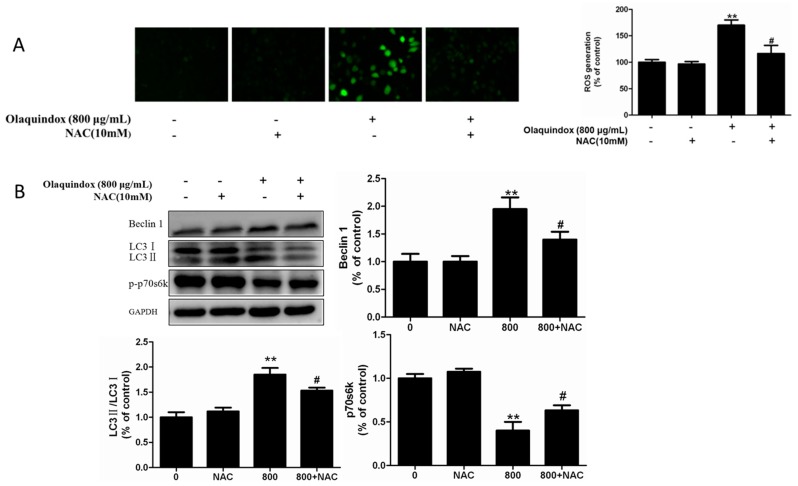
Effect of reduce ROS level on olaquindox induced autophagy. (**A**) NAC alleviated olaquindox-induced generation of ROS. ROS was assessed as described in Figure 2A; (**B**) Cells were pretreated with NAC (10 mM) and the expression of Beclin 1, LC3II/LC3I and p70s6k. All data represents means ± SD from three independent experiments. ** *p* < 0.01, compared to the control group; ^#^ *p* < 0.05, compared to the olaquindox alone group.

The ROS scavenger NAC was used to demonstrate the role of ROS generation in autophagy. The results showed that pretreatment with NAC could effectively block the ROS generation caused by olaquindox treatment (Figure 4A). Meanwhile, pretreatment with NAC could alleviate olaquindox-induced autophagy as proved by the decreased expression of Beclin1 and LC3II/LC3I and increased the expression of phosphorylation-p70s6k (Figure 4B), compared to the olaquindox alone group.

### 2.5. Effect of TSC2 on Olaquindox-Induced Autophagy

**Figure 5:**
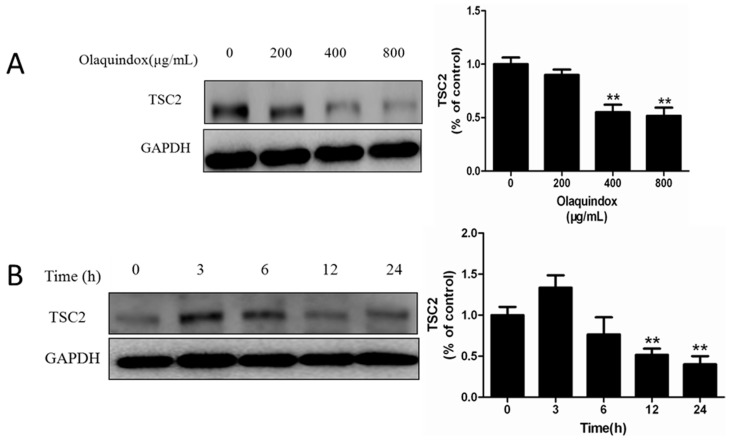

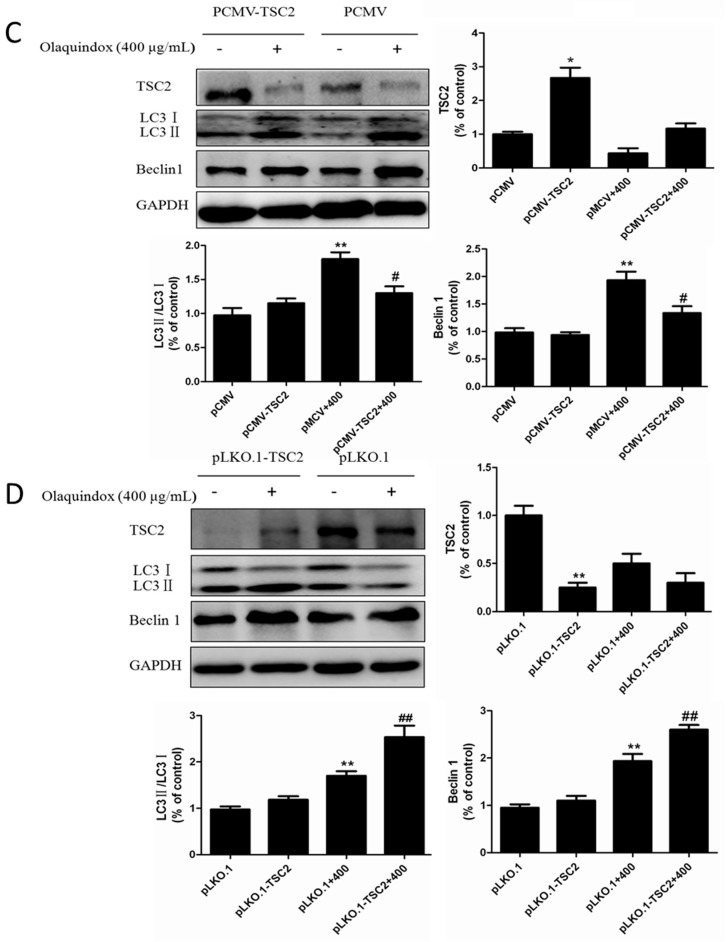
Effect of TSC2 on olaquindox-induced autophagy. (**A**) Expression of TSC2 treated with olaquindox (0, 200, 400, 800 μg/mL) for 24 h was detected by western blotting analysis; (**B**) Cells treated with 800 μg/mL olaquindox for different time points (0–24 h) and expression of TSC2 was detected by western blotting; (**C**) Overexpression of TSC2 inhibited olaquindox-induced the expression of LC3II/LC3I and Beclin 1; (**D**) Knockdown of TSC2 enhanced olaquindox-induced the expression of LC3II/LC3I and Beclin 1. GAPDH was used for loading control. All data represents means ± SD from three independent experiments. * *p* < 0.05, ** *p* < 0.01, compared to the control pCMV or pLKO.1 transfected cells; ^#^ *p* < 0.05, ^##^ *p* < 0.01, compared to olaquindox-treated pCMV or pLKO.1 transfected cells.

As shown in Figure 5A,B, olaquindox could decrease the expression of TSC2 in HEK293 cells in a dose and time-dependent manner. To further determine the role of TSC2 in olaquindox-induced autophagy, TSC2 interference plasmid and overexpression plasmid were used. After transfecting the pCMV-TSC2 plasmid, the expression of TSC2 successfully increased to 2.8-fold (*p* < 0.01), compared to pCMV cells (Figure 5C). Consistently, pLKO.1-TSC2 transfection effectively reduced TSC2 protein expression to 0.32-fold (*p* < 0.01), compared with control pLKO.1 cells (Figure 5D). Overexpression of TSC2 in olaquindox-treated HEK293 cells could significantly reduce the expression of LC3II/LC3I and Beclin 1, compared with the transfected control group induced by olaquindox (Figure 5C). In contrast, knockdown of TSC2 in HEK293 cells increased olaquindox-induced autophagy characterized by increased expression of LC3II/LC3I and Beclin 1 (Figure 5D). The result indicated that TSC2 acted as a negative regulator of autophagy in olaquindox treatment.

### 2.6. Effects of TSC2 on Olaquindox-Induced ROS Generation and Oxidative Stress in HEK293 Cells

**Figure 6:**
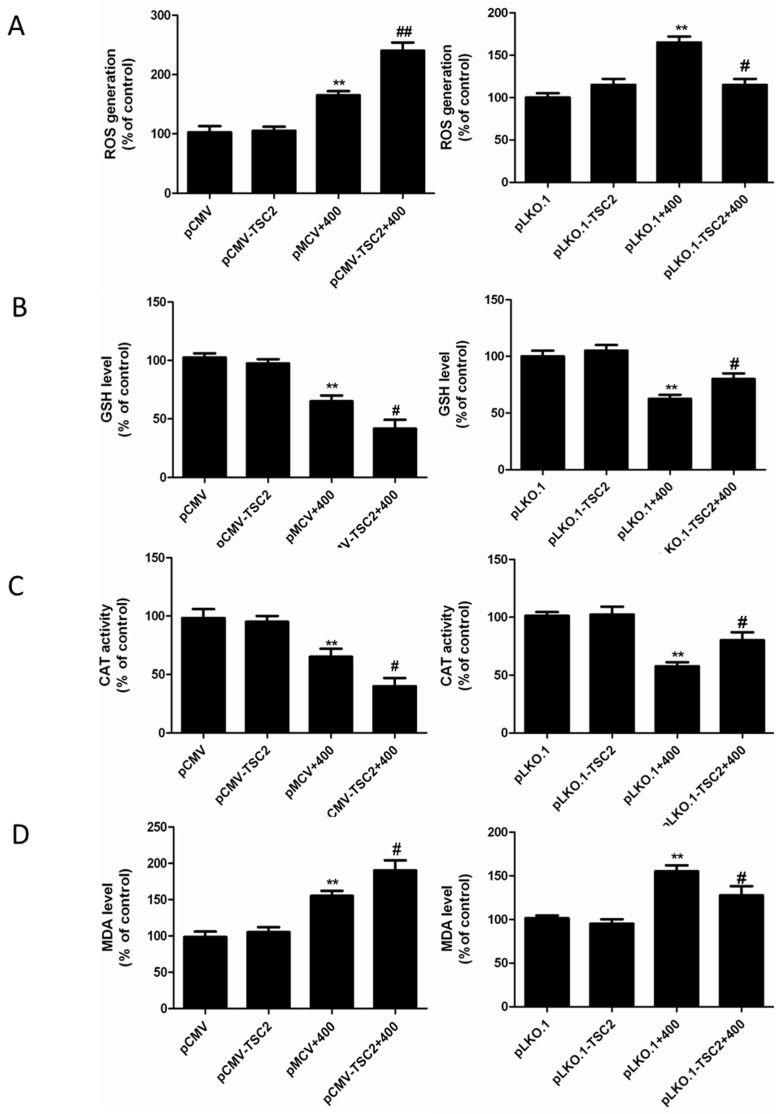
Effects of TSC2 on olaquindox-induced ROS generation and oxidative stress in HEK293 cells. (**A**) Intracellular ROS production was measured by 2,7-dichlorofluorescein diacetate (DCFH-DA) in TSC2 overexpressed and knockdown cells. (**B**–**D**) Effect of olaquindox treated on cellular glutathione (GSH) levels, catalase (CAT) activities and malondialdehyde levels (MDA) in TSC2 overexpressed and knockdown cells, respectively. ** *p* < 0.01, compared to the control pCMV or pLKO.1 transfected cells; ^#^ *p* < 0.05, ^##^ *p* < 0.01, compared to olaquindox-treated pCMV or pLKO.1 transfected cells.

The result showed that overexpression of TSC2 enhanced olaquindox-induced ROS generation (Figure 6A). Consistently, in the TSC2 knockdown cells, lower levels of ROS were detected (Figure 6A). To further explore the function of TSC2 in olaquindox-induced oxidative stress, we examined the levels of GSH, CAT and MDA.

As shown in Figure 6B–D, overexpression of TSC2 in olaquindox- treated 293 cells increased the levels of MDA and decreased the activity of GSH and CAT, compared to transfection of pCMV cells by olaquindox treatment. Meanwhile, knockdown of TSC2 in HEK293 cells increased the activity of GSH and CAT and diminished the levels of MDA, compared to pLKO.1 transfected cells by olaquindox treatment. The results indicated that TSC2 could promote the olaquindox-induced ROS production and oxidative damage in HEK293 cells.

### 2.7. Effect of TSC2 on Olaquindox-Induced Apoptosis

**Figure 7:**
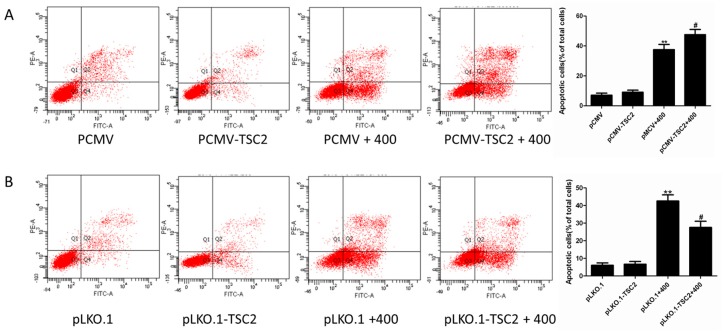
Effect of TSC2 on olaquindox-induced apoptosis. (**A**) Increased of olaquindox-induced apoptosis by overexpressed TSC2 in 293 cells was estimated by flow cytometry with Annexin V-FITC/PI staining (**B**) Reduced of olaquindox-induced apoptosis by knockdown of TSC2 in 293 cells was estimated by flow cytometry with Annexin V-FITC/PI staining. All data represents means ± SD from three independent experiments. ** *p* < 0.01, compared to the control pCMV or pLKO.1 transfected cells; ^#^ *p* < 0.05, compared to olaquindox-treated pCMV or pLKO.1 transfected cells.

As shown in Figure 7A, after olaquindox treatment, overexpression of TSC2 in HEK293 cells increased the apoptotic cells from 37.4% to 49.6% (*p* < 0.05), compared to that of PCMV cells induced by olaquindox. On the contrary, interference with the expression of TSC2 in olaquindox-treated HEK293 cells reduced the apoptotic cells from 39.6% to 29.2% (*p* < 0.05), compared to that of pLKO.1 cells induced by olaquindox (Figure 7B).The results indicated that TSC2 played a pro-apoptotic function in olaquindox-induced apoptosis.

## 3. Discussion

Olaquindox is used as a feed additive, but the toxic effects of olaquindox have drawn public attention. A large number of animal studies have revealed that oxidative stress damage in mice tissues, including liver, kidney and adrenal gland, could be caused by treatment with QdNOs such as olaquindox, quinocetone, carbadox and their metabolites [7,21,22]. Nevertheless, molecular mechanism research in an in vitro model is particularly important. In our previous studies, it has been demonstrated that olaquindox could induce apoptosis, DNA damage, S-phase arrest and autophagy in HepG2 cells [3,9,10]. Therefore, in this research, HEK293 cells treated with olaquindox were used as a toxicity model in order to evaluate the renal toxicity in vitro, which is necessary for the safety evaluation of olaquindox. In this process, we investigated the cytotoxicity of olaquindox and the role of TSC2 in olaquindox-induced autophagy in HEK293 cells.

Our results revealed that olaquindox treatment of HEK293 cells caused cytotoxicity and the corresponding IC_50_ value was approximately 800 μg/mL (Figure 1A), which was consistent with the previous studies [23]. The IC_50_ of HEK293 cells is similar to that of HepG2 cells treated with olaquindox [3]. Morphological changes could be observed in HEK293 cells exposed to olaquindox (Figure 1B,D). Furthermore, the caspase-3/7 activity was increased in a dose-dependent manner by olaquindox treatment. Similar results were also demonstrated in other cell types, including HepG2 [3], indicating that olaquindox could induce cell apoptosis. It has been reported that the main metabolic pathway of QdNOs was the N-oxide group reduction, evidenced by the fact their activities and toxicities are often diminished through this N-oxide reduction [21].

Excess generation of ROS contributes to lipid peroxidation and malonaldehyde, which is one of the most apparent biological markers of oxidative stress damage [24]. In the current study, the results showed that olaquindox treatment significantly enhanced ROS production and the levels of MDA were increased in HEK293 cells. Meanwhile, the activity of the antioxidant enzymes CAT and GSH levels decreased (Figure 2). In short, these findings indicate that olaquindox could not only induce ROS generation directly, but also decrease the cells’ ability to break oxygen radical chains, further aggravating ROS-mediated oxidative damage.

LC3 is a biomarker of the existence of autophagosomes and LC3-I undergoes cleavage and is converted to a processed form (LC3-II) during autophagy [25]. Beclin 1, an important autophagy protein, could complement the defects present in autophagy [26]. p70s6k is a mTOR pathway effector and accumulated evidence suggests that mTOR/p70s6k signaling contributes to autophagy [27]. p70s6k is a key translation regulator which can be directly phosphorylated by the mTOR pathway, a negative regulator of autophagy [28]. In our study, more MDC-labeled particles and higher fluorescent density were observed in 293 cells treated with olaquindox (Figure 3A). Meanwhile, olaquindox treatment markedly increased the expression levels of Beclin 1 and LC3II/LC3I, and decreased phosphorylation p70S6K in a dose and time-dependent manner, which suggested olaquindox-induced autophagy in HEK293 cells. Recently, numerous studies have shown that ROS could regulate the autophagy process [29,30]. Afterwards, we examined whether the production of ROS had an impact on autophagy caused by olaquindox treatment. Nevertheless, the current results revealed that pretreatment with NAC efficiently reduced ROS generation and blocked olaquindox-induced autophagy as well (Figure 4). However, there are a number of reports related to inhibition of ROS-mediated autophagy [31,32]. In short, we speculate that ROS might play an upstream role in olaquindox-induced autophagy.

TSC2 has been proved to be closely associated with autophagy. It has been demonstrated that oxidative stress-induced Tnfaip8 l1/Oxi-β stabilizes TSC2 protein, decreases the expression of phosphorylation mTOR, and increases autophagy [33]. Another study showed that inhibition of mTORC1 by rapamycin activated autophagy and subsequently rescued TSC2 knockout cells [34]. In the current study, olaquindox could decrease the expression of TSC2 in a dose and time-dependent manner in HEK293 cells (Figure 5A,B). This result indicates that TSC2 might play a negative control role in the olaquindox-induced autophagy pathway. Thus, we tried to clarify the effect of TSC2 in olaquindox-induced autophagy. Interestingly, the results showed that overexpression of TSC2 reduced the expression of Beclin 1 and LC3II/LC3I (Figure 5C). On the contrary, suppression of TSC2 enhanced the expression of Beclin 1 and LC3II/LC3I (Figure 5D). Our results reveal that TSC2 plays an anti-autophagic role and olaquindox induced autophagy by reducing TSC2 expression in HEK293 cells. Generally speaking, TSC2 exerts its tumor suppressor function through negative regulation mTOR pathways which negatively regulate autophagy [35,36]. TSC2 is bound by peroxisomal biogenesis factors 5 (PEX5), and peroxisome-localized TSC functions as a RhebGTPase-activating protein (GAP) to suppress mTOR and prompt autophagy [37]. Data has demonstrated that knockout of TSC2 resulted in autophagic activity involved with AMPK-dependent activation of ULK1 [20]. Thus, TSC2 may participate in negative regulation of olaquindox-induced autophagy.

Next, we tried to illuminate the relationship between ROS and TSC2. Up-regulation of TSC2 enhanced olaquindox-induced ROS production, whereas down-regulation of TSC2 attenuated the ROS production (Figure 6). These results revealed that TSC2 could promote the olaquindox-induced ROS production and oxidative damage in HEK293 cells. TSC2 could enhance oxidative stress damage by means of regulating the Nrf2 signaling pathway. A number of signaling molecules operate both upstream and downstream of mTORC1/TSC2 as well as of Nrf2. Data showed that Sestrins 2 could decrease ROS levels both by inhibiting mTORC1 and by inducing Keap1 degradation and Nrf2 activation, given that p62 is upregulated as a result of the inhibition of autophagy by mTORC1 [38]. In the present study, the results showed that overexpression of TSC2 increased olaquindox-induced apoptosis (Figure 7A) whereas suppression of TSC2 attenuated olaquindox-induced apoptosis in 293 cells (Figure 7B). The results indicated that TSC2 played a pro-apoptotic function in olaquindox-induced apoptosis. Knockdown of TSC2 induced synergistic cell death which was different in cancer cells, as no significant cell death effect was found in vascular smooth muscle cells after knockdown of TSC2 [39]. Treatment with olaquindox induced cell apoptosis, as well as autophagy. In most instances, autophagy appears to promote cell survival by blocking apoptotic cell death [10]. Autophagy is a protective response against advanced glycation end product-induced apoptosis in mesangial cells [12]. We inferred that autophagy might act as a self-defense mechanism in HEK293 cells exposed to olaquindox treatment. However, further studies are required to reveal the details.

## 4. Materials and Methods

### 4.1. Materials

Olaquindox (purity ≥ 98%, CAS No.23696-28-8) was purchased from the China Institute of Veterinary Drug Control (Beijing, China). Dulbecco’s modified Eagle’s medium (DMEM) was purchased from Invitrogen (Gibco, Grand Island, NY, USA). Fetal bovine serum (FBS) was obtained from Thermo Fisher (Beijing, China). *N*-Acetylcysteine (NAC), monodansylcadaverin (MDC) and 3-(4,5-dimethyl-2-thiazolyl)-2,5-diphenyl-2*H*-tetrazolium bromide (MTT), were acquired from Sigma–Aldrich (St. Louis, MO, USA). Trypsin and dimethyl sulfoxide (DMSO) and sodium dodecylsulfonate (SDS) were all bought from AMRESCO Inc. (Solon, OH, USA). All other reagents were of analytical grade.

### 4.2. Cell Culture

The HEK293 cell line was obtained from the American Type Culture Collection (Manassas, VA, USA). Cells were cultured in DMEM containing 10% fetal bovine serum, 1% penicillin and streptomycin (Beyotime Institute of Biotechnology, Haimen, China) at 37 °C in a wetted atmosphere of 5% CO_2_. According to our previous study, olaquiadox was dissolved in DMEM to make final concentrations of 200, 400, 800 μg/mL [3].

### 4.3. Plasmid Transfection

The TSC2 interference plasmid pLKO.1-TSC2 and TSC2 expression plasmid PCMV-TSC2 were purchased from Addgene (Cambridge, MA, USA). The vector plasmids (pLKO.1 and PCMV) carrying a non-targeted sequence were used as control. As described by the manufacturer, HEK293 cells (1 × 10^5^ cells/well) grown on six-well plates were transfected with 2 μg of plasmid using 6 mL of X-tremeGENE HP DNA transfection reagent (Roche, Basel, Switzerland). After 48 h, the cells were harvested to performed experiments.

### 4.4. Cell Viability Assay

The cell viability was evaluated by MTT assay as previous study [40]. In brief, HEK293 cells (2 × 10^4^ cells per well) were plated in a 96-well plate with a final volume of 100 mL DMEM. After olaquindox treatment for 24 h, the medium was replaced by 100 μL fresh DMEM containing 0.5 mg/mL MTT solution. After incubated for 4 h in the dark, the culture solution was removed and 100 μL DMSO was added for 15 min. The optical density was measured by a microplate reader at 570 nm (Molecular Devices, Sunnyvale, CA, USA).

### 4.5. Analysis of Apoptosis

According to our previous study, cell apoptosis was performed using Hoechst 33342 staining [41]. In short, HEK293 cells (1 × 10^5^ cells/well) were seeded into 6-well culture plates and treated with olaquindox (0, 200, 400, 800 μg/mL) for 24 h. Then, cells were added 1 mL DMEM containing 1 mg/mL Hoechst 33342 (Vigorous Biotechnology, Beijing, China). After culturing for 30 min in the dark, cells were examined under a fluorescence microscope (Leica Microsystems, Wetzlar, Germany). Cell chromatin condensation was indicated as apoptotic cells. We used an annexin V-FITC apoptosis detection kit (Vazyme Biotech Co., Ltd., Nanjing, China) for flow cytometric analysis of apoptosis. Cells were collected by 0.65% trypsin without EDTA. Cells were resuspended in 500 μL binding buffer after washed twice with PBS. Eventually, cells were added into 5 μL annexin V-FITC and 5 μL propidium iodide for 10 min. Data was analyzed by BD FACSAria™ flow cytometer (BD Biosciences, San Jose, CA, USA).

### 4.6. Caspase-3/7 Activity Examination

Caspase-3/7 activity after olaquindox treatment was measured by the Caspase-Glo^®^ 3/7 assay kit (Promega Corp., Madison, WI, USA). Briefly, HEK293 cells (2 × 10^4^ cells/well) were cultured in 96-well plates for 24 h and then treated with olaquindox for additional 24 h. Afterwards, cells were added 200 μL Caspase-Glo^®^ 3/7 solution per well for 1 h in the dark. Optical density was recorded by a fluorophotometer (Molecular Devices).

### 4.7. Intracellular ROS Examination

The intracellular ROS production was detected by fluorescent dye DCFH-DA (Beyotime Institute of Biotechnology). HEK293 cells (1 × 10^5^ cells/well) were seeded in 6-well plates for 24 h. The cells were treated with 100 mL DMEM with olaquindox at different final concentrations for 24 h. After exposure to olaquindox, the cells were stained with 10 μmol/L DCFH-DA for 20 min. Then, cells were washed three times with PBS and imaged by a fluorescent microscope (Leica Microsystems). The fluorescence was detected by a multimode plate reader (Thermo Fisher Scientific, Bremen, Germany).

### 4.8. Intracellular Glutathione (GSH), Catalase (CAT) and Malondialdehyde (MDA) Examination

The levels of GSH, CAT and MDA were determined by specific assay kits (Nanjing Jiancheng Nanjing, China). HEK293 cells (1 × 10^5^ cells/well) were seeded into 12-well plates and then treated with olaquindox (0, 200, 400 and 800 μg/mL) for an additional 24 h. According to the manufacturer’s instructions, cells were lysed using the cell lysis buffer supplied with the assay kits. The cell lysates were centrifuged at 14,000 rpm for 10 min at 4 °C. The concentrations of proteins were calculated using the BCA protein assay kit (Beyotime Institute of Biotechnology).

### 4.9. Monodansylcadaverine (MDC) Staining Assay

MDC, a fluorescent dye, is commonly used as an effective indicator for autophagosome. HEK293 cells were cultured into 6-well plates and exposed to different concentrations (0, 200, 400 and 800 μg/mL) of olaquindox for 24 h. Then, cells were incubated with medium containing 50 μM MDC for 30 min. Then autophagy was observed by a fluorescence microscope (Leica Microsystems).

### 4.10. Western Blotting Analysis

Western blotting was performed according to our previous study [42]. After treatment with olaquindox, cells were collected and lysed in a lysis buffer containing 20 mMTris–HCl, 4% SDS, 1 mM EDTA, 50 mM NaF, 0.5 mM Na_3_VO_4_ and 1 mM PMSF at 4 °C for 15 min. Protein in the buffer was loaded into SDS-polyacrylamide gel (SDS-PAGE) for electrophoresis. Then, running gel was transported to nitrocellulose membranes. After being blocked with 5% non-fat milk for 2 h, the membranes were washed with tris buffered saline tween (TBST) and incubated with primary and secondary antibodies. Finally, the membranes were measured by ECL luminescent detection kit (Vigorous Biotechnology). Western blot density was evaluated by the ImageJ 1.46 software (National Institutes of Health, Bethesda, MD, USA). The primary antibodies were performed as followed: rabbit polyclonal antibodies against TSC2, LC3, phosphorylation-p70s6k (p70s6k) (1:1000; Santa Cruz Biotechnology, Inc., Santa Cruz, CA, USA) and Beclin 1 (1:1000; ABclonal Biotech, Cambridge, MA, USA), mouse polyclonal antibodies against GAPDH (1:2000; Zhongshan Golden Bridge, Beijing, China). The secondary antibodies were anti-rabbit IgG (1:5000) and anti-mouse IgG (1:5000) (Zhongshan Golden Bridge).

### 4.11. Statistical Analysis

Histogram analysis was completed using Graph Pad Prism 5.0 (Graph Pad Software, La Jolla, CA, USA). Results were expressed as means ± SD from three independent experiments. Statistical analysis were carry out by SPSS V13.0 (SPSS Inc., Chicago, IL, USA) with one-way analysis of variance (ANOVA), followed by the LSD post hoc test. A *p* <0.05 was considered to be significant.

## 5. Conclusions

In conclusion, our present study revealed that treatment with olaquindox induced ROS generation and cell apoptosis, as well as autophagy in HEK293 cells, which the TSC2 pathway partly participated in it. Importantly, TSC2 was involved in the negative regulation of olaquindox-induced autophagy in HEK293 cells, which may offer a novel understanding of the toxicity of olaquindox or other QdNOs.
